# Variance of the root mean square value of the residuals of sine fitting in the presence of additive noise

**DOI:** 10.1038/s41598-025-32688-2

**Published:** 2025-12-21

**Authors:** Francisco A. C. Alegria

**Affiliations:** Instituto de Telecomunicações and Instituto Superior Técnico of the University of Lisbon, Lisbon, Portugal

**Keywords:** Sine-fitting, Least-squares, Estimation uncertainty, Additive noise., Engineering, Mathematics and computing, Physics

## Abstract

The least-squares fitting of a sinusoidal model to a set of data points is a common procedure in signal processing algorithms. A residual is the difference between the value of one data points and the estimated value of that point given by the sinusoidal model. The root mean square (RMS) value of all the residuals is a common metric used in many applications to quantify the goodness of fit. In analog-to-digital conversion, for example, the RMS value is used to compute the number of effective bits. In other applications the RMS value is used to compute the signal-to-noise ratio which measures the amount of noise generated by an electronic circuit such as an amplifier, for instance. Due to the presence of different random non-ideal phenomena affecting the data points, like stimulus signal phase noise, sampling jitter or quantization error, the estimative of the RMS value is uncertain and whose statistical properties are important to evaluate. In this work we focus on the effect that additive noise has on the variance of the RMS value of the residuals. A first exact analytical expression is derived and two easier to use and simpler approximations are proposed. The results presented are validated using numerical simulations employing a Monte Carlo type procedure.

## Introduction

The least squares fitting of sinusoidal models to experimental data represents one of the most fundamental and widely applied procedures in modern signal processing and measurement science. This mathematical technique, which seeks to minimize the sum of squared differences between observed data points and a theoretical sinusoidal function, has found extensive applications across diverse fields ranging from analog-to-digital converter (ADC) characterization to electronic circuit noise analysis^1,2^. At the heart of this fitting procedure lies the concept of residuals—the differences between actual measurements and the values predicted by the fitted sinusoidal model. The root mean square (RMS) value of these residuals serves as a critical metric for quantifying the goodness of fit and, consequently, the quality of the underlying measurement system or signal processing algorithm.

The importance of RMS residual analysis extends far beyond simple curve fitting applications. In the realm of ADC testing, for instance, the RMS value of residuals obtained from sinusoidal fitting directly determines the effective number of bits (ENOB), a fundamental performance parameter that characterizes the dynamic accuracy of analog-to-digital conversion systems^3,4^. The IEEE Standard 1241–2010 explicitly defines standardized procedures for ADC testing that rely heavily on least squares sine wave fitting algorithms, where the power of the residual signal becomes the primary indicator of converter performance^5^. Similarly, in amplifier characterization and electronic circuit design, RMS residual values are routinely employed to compute signal-to-noise ratios (SNR), which quantify the amount of unwanted noise generated by electronic components and systems.

However, the statistical properties of RMS residual estimates are far from trivial, particularly when the underlying data is contaminated by various sources of random noise and systematic errors. Real-world measurement scenarios invariably involve multiple non-ideal phenomena that can significantly affect the accuracy and reliability of RMS residual calculations. These include, but are not limited to, stimulus signal phase noise, sampling jitter in data acquisition systems, quantization errors inherent in digital measurement instruments, and additive thermal noise from electronic components^6,7^. Each of these error sources introduces uncertainty into the parameter estimation process, which subsequently propagates through to the final RMS residual calculation, making the statistical characterization of these estimates a matter of considerable practical importance.

Recent advances in signal processing theory have highlighted the need for more sophisticated analytical frameworks for understanding the statistical behavior of parameter estimation algorithms under realistic noise conditions^8,9^. Traditional approaches to residual analysis often assume idealized conditions, such as white Gaussian noise with known statistical properties, which may not accurately reflect the complex noise environments encountered in practical applications. Modern measurement systems frequently operate in scenarios where multiple noise sources interact in non-trivial ways, where noise characteristics may be frequency-dependent, or where the underlying signal model itself may be subject to uncertainty^10^.

The theoretical foundation for understanding RMS residual variance in sinusoidal fitting can be traced back to fundamental principles of least squares estimation theory and the statistical properties of parameter estimators^11^. When a sinusoidal model is fitted to noisy data using least squares techniques, the resulting parameter estimates are themselves random variables whose statistical properties depend on the characteristics of the underlying noise processes. The residuals computed from these parameter estimates inherit this randomness, leading to variability in the calculated RMS values that must be properly characterized for meaningful interpretation of measurement results.

Contemporary research in this area has focused on developing more accurate analytical expressions for the variance of RMS residual estimates, particularly in the presence of additive noise with known statistical properties. These efforts have been motivated by the recognition that existing approximations, while useful for preliminary analysis, may not provide sufficient accuracy for demanding applications such as high-precision ADC testing or low-noise amplifier characterization. The development of exact analytical expressions, complemented by simpler approximations suitable for routine use, represents a significant advancement in the theoretical understanding of these fundamental measurement procedures.

The practical implications of improved RMS residual variance models extend across multiple domains of engineering and scientific measurement. In the field of ADC testing, more accurate variance estimates enable better discrimination between genuine performance differences and measurement uncertainty, leading to more reliable product qualification and improved manufacturing yield. For amplifier and circuit designers, enhanced understanding of RMS residual statistics facilitates more accurate noise budgeting and performance prediction, ultimately contributing to the development of higher-performance electronic systems. Additionally, in research applications where sinusoidal fitting is used for parameter extraction from experimental data, improved statistical models enable more rigorous uncertainty analysis and more confident interpretation of results^12^.

The validation of theoretical models for RMS residual variance typically relies on extensive numerical simulations employing Monte Carlo techniques. These simulations involve generating large numbers of synthetic datasets with known statistical properties, applying the sinusoidal fitting algorithms under investigation, and comparing the observed statistical behavior of RMS residuals with theoretical predictions. The agreement between simulation results and analytical models serves as a crucial test of model validity and provides confidence in the practical applicability of the theoretical framework.

The novelty of the present study lies in the fact that, unlike the previous analyses carried out by Alegria^13,14^, which examined the expected value and the variance of the RMS of sine-fitting residuals in the presence of phase noise or sampling jitter, the current work investigates the same statistical quantity under a fundamentally different noise mechanism — additive amplitude noise. While timing-related perturbations such as phase noise and jitter primarily affect the temporal positions of the samples, additive noise directly alters their amplitude values. Consequently, the analytical results presented here extend the theoretical framework established by Alegria^13,14^ to a new and practically important class of noise sources, providing a more complete statistical characterization of sine-fitting residuals relevant to measurement and signal-processing applications.

In the published literature, many different sine-fitting techniques are available for sinusoidal fitting models. The classical three-parameter least-squares (LS) model generally estimates amplitude, phase, and offset with the assumption that the frequency of the signal is known. A four-parameter LS version models four parameters, adding frequency as an extra parameter typically solved by iterative optimization. Other parameter estimation techniques—known as maximum likelihood, total least squares, or frequency-domain estimation—have also been published to achieve more robustness or improved computation time under certain conditions. The effect of the characteristics of noise and disturbance on robustness of sine-fitting analysis has been demonstrated for additive Gaussian noise, quantization noise, and phase or sampling jitter, as all disturbances affect the residual statistics differently. This paper will consider additive white Gaussian noise, which is still the most common and theoretically adequate model. The overall form of the derivations provided in this paper are applicable to other zero-mean noise processes of finite variance and the properties regarding the RMS (root mean squares) variances of the residual are likely to carry over qualitatively for a wide range of uncorrelated noise processes.

This work addresses the existing gap in the literature concerning the analytical modeling of the variance of the RMS of sine-fitting residuals in the presence of additive amplitude noise. Although prior studies have derived expressions for the expected value and variance of this quantity under phase noise and sampling jitter^13,14^, no exact theoretical formulation has been available for the additive-noise case, which is the most common disturbance in practical measurement systems. The present paper fills this gap by deriving an exact analytical expression for this variance, proposing simpler closed-form approximations suitable for practical use, and validating the results through extensive Monte Carlo simulations. The research focuses specifically on the effects of additive noise on the variance of RMS residual values, providing both fundamental insights into the underlying statistical mechanisms and practical tools for improved measurement uncertainty analysis. The theoretical developments are validated through comprehensive Monte Carlo simulations that demonstrate the accuracy and practical utility of the proposed analytical framework.

## Analytical derivation

In this section we present the analytical derivation of an expression that allows us to determine the standard deviation of the RMS value of the sinefitting residuals.

The following symbols are used throughout the paper:

- $\:A$– amplitude of the fitted sinusoid (V).
- $\:\varphi\:$– phase of the fitted sinusoid (rad).
- $\:{f}_{x}$– signal (excitation) frequency (Hz).
- $\:{f}_{s}$– sampling frequency (Hz).
- $\:M$– number of acquired samples.
- $\:{n}_{i}$– additive noise sample, assumed zero-mean Gaussian with variance $\:{\sigma\:}_{n}^{2}$.
- $\:{\sigma\:}_{n}$– standard deviation of the additive noise (V).
- $\:{x}_{i}$– measured data sample.
- $\:{\widehat{x}}_{i}$– reconstructed sample from the fitted sinusoid.
- $\:SSR$– sum of squared residuals.
- $\:RMS$– root-mean-square of the residuals.
- $\:\nu\:$– number of degrees of freedom ($\:M-3$).
- $\:{{\Omega\:}}_{0}=2\pi\:{f}_{x}/{f}_{s}$– normalized angular frequency.
- $\:\alpha\:$– significance level for the confidence interval.
- $\:R$–number of repetitions of the Monte-Carlo type procedure.

The first step is, naturally, to start with the sinusoidal model. We thus express the ideal sample values using

1 $$\:{y}_{i}=C+A\cdot\:\mathrm{cos}\left({\omega\:}_{x}{t}_{i}+\phi\:\right)$$,

where $\:C$ is the sinusoidal offset, $\:A$ is the amplitude and $\:\phi\:$ is the initial phase. The sampling instants are given by $\:{t}_{i}$, where the sample index, $\:i$ goes from 0 to $\:M-1$ where $\:M$ is the number of samples. These instants are equally spaced in time by an interval which is the reciprocal of the sampling frequency $\:{f}_{s}$. The sinusoidal frequency is assumed known and is thus not estimated in the context of this work. It is given by $\:{f}_{x}$ and the corresponding angular frequency is given by $\:{\omega\:}_{x}$.

Here we study the case where there is additive noise affecting the sample values, so that the actual samples are given by

2 $$\:{z}_{i}=C+A\cdot\:\mathrm{cos}\left({\omega\:}_{x}{t}_{i}+\phi\:\right)+{n}_{i}$$,

where $\:{n}_{i}$ is the amount of additive noise that affects sample $\:i$.

In the following derivations, the additive noise $\:{n}_{i}$ is assumed to be a zero-mean, statistically independent, and identically distributed random variable with variance $\:{\sigma\:}_{n}^{2}$. In accordance with standard practice in measurement science and signal processing, $\:{n}_{i}$ is modeled as a Gaussian (normally distributed) random variable, i.e., $\:{n}_{i}\sim\:\mathcal{N}(0,{\sigma\:}_{n}^{2})$^15,16^. This assumption ensures that the residuals after least-squares sine fitting are also normally distributed, which justifies the use of chi-squared statistical properties in the subsequent analytical developments.

Given this set of data points, corrupted by noise, we wish to estimate the three unknown parameters that describe the sinusoid – offset ($\:\widehat{C}$), amplitude ($\:\widehat{A}$) and initial phase ($\:\widehat{\phi\:}$). Note the “hat” symbol over the estimated quantities. We can thus use a three-parameter sinefitting algorithm which minimizes the square difference between the data points and the sinusoidal model, that is, it minimizes the RMS error given by

3 $$\:\widehat{RMS}=\sqrt{\frac{1}{M}\sum\:_{i=0}^{M-1}{\left({z}_{i}-{\widehat{z}}_{i}\right)}^{2}}$$,

where the estimated sinusoidal points are given by

4 $$\:{\widehat{z}}_{i}=\widehat{C}+\widehat{A}\cdot\:\mathrm{cos}\left({\omega\:}_{x}\cdot\:{t}_{i}+\widehat{\phi\:}\right)$$.

It will be useful to introduce the sum of squared residuals given by

5 $$\:\widehat{SSR}=\sum\:_{i=0}^{M-1}{\left({z}_{i}-{\widehat{z}}_{i}\right)}^{2}$$,

such that the estimated root mean square value of the residuals, given by (3), can be expressed as

6 $$\:\widehat{RMS}=\sqrt{\frac{\widehat{SSR}}{M}}$$.

The variance of this estimator can be obtained by subtracting the square of the mean from the second raw moment,

7 $$\:\mathrm{v}\mathrm{a}\mathrm{r}\left\{\widehat{RMS}\right\}=\mathrm{E}\left\{{\widehat{RMS}}^{2}\right\}-{\mathrm{E}}^{2}\left\{\widehat{RMS}\right\}$$.

Under the assumption of additive zero-mean noise with variance $\:{\sigma\:}_{n}^{2}$, the expected value of the estimated sum of squared residuals (SSR) obtained from the three-parameter sine fit is

8 $$\:\mathbb{E}\left[SSR\right]=(M-3){\sigma\:}_{n}^{2}$$,

where $\:M$is the number of data points and 3 is the number of fitted parameters (offset, amplitude, and phase). This result follows directly from classical least-squares estimation theory for linear models^17^.

Using this result together with Eq. (6) leads to the second raw moment of $\:\widehat{RMS}$,

9 $$\:\mathrm{E}\left\{{\widehat{RMS}}^{2}\right\}=\frac{M-3}{M}\cdot\:{\sigma\:}_{n}^{2}$$.

Focusing now on the expected value of $\:\widehat{RMS}$ we see, from (6), since $\:M$ is a constant, that

10 $$\:\mathrm{E}\left\{\widehat{RMS}\right\}=\sqrt{\frac{1}{M}}\cdot\:\mathrm{E}\left\{\sqrt{\widehat{SSR}}\right\}$$.

Multiplying and dividing by the additive noise variance leads to

11 $$\:\mathrm{E}\left\{\widehat{RMS}\right\}=\sqrt{\frac{1}{M}}\cdot\:{\sigma\:}_{n}\cdot\:\mathrm{E}\left\{\sqrt{\frac{\widehat{SSR}}{{\sigma\:}_{n}^{2}}}\right\}$$.

The variable $\:\frac{\widehat{SSR}}{{\sigma\:}_{n}^{2}}$ has a chi-squared distribution, $\:{{\upchi\:}}^{2}\left(\nu\:\right)$, where $\:\nu\:=M-3$ represents the number of degrees of freedom. It thus follows that

12 $$\:\mathrm{E}\left\{\widehat{RMS}\right\}={\sigma\:}_{n}\cdot\:\sqrt{\frac{1}{M}}\cdot\:\mathrm{E}\left\{\sqrt{{{\upchi\:}}^{2}\left(M-3\right)}\right\}$$.

The expected value of the square root of a chi-squared random variable with $\:k$ degrees of freedom is given by

13 $$\:\mathrm{E}\left\{\sqrt{{\chi\:}^{2}}\right\}=\sqrt{2}\cdot\:\frac{{\Gamma\:}\left(\frac{k+1}{2}\right)}{{\Gamma\:}\left(\frac{k}{2}\right)}$$.

We thus have

14 $$\:\mathrm{E}\left\{\widehat{RMS}\right\}={\sigma\:}_{n}\cdot\:\sqrt{\frac{2}{M}}\cdot\:\frac{{\Gamma\:}\left(\frac{M-2}{2}\right)}{{\Gamma\:}\left(\frac{M-3}{2}\right)}$$.

Using (10) leads to

15 $$\:\mathrm{E}\left\{\widehat{RMS}\right\}={\sigma\:}_{n}\cdot\:\sqrt{\frac{2}{M}}\cdot\:\frac{{\Gamma\:}\left(\frac{M-2}{2}\right)}{{\Gamma\:}\left(\frac{M-3}{2}\right)}$$.

Inserting this, and (9), into (7) leads to

16 $$\:\mathrm{v}\mathrm{a}\mathrm{r}\left\{\widehat{RMS}\right\}=\frac{M-3}{M}\cdot\:{\sigma\:}_{n}^{2}-{\left[{\sigma\:}_{n}\cdot\:\sqrt{\frac{2}{M}}\cdot\:\frac{{\Gamma\:}\left(\frac{M-2}{2}\right)}{{\Gamma\:}\left(\frac{M-3}{2}\right)}\right]}^{2}$$,

which can be written as

17 $$\:\mathrm{v}\mathrm{a}\mathrm{r}\left\{\widehat{RMS}\right\}={\sigma\:}_{n}^{2}\cdot\:\left[\frac{M-3}{M}-\frac{2}{M}\cdot\:\frac{{{\Gamma\:}}^{2\left(\frac{M-2}{2}\right)}}{{{\Gamma\:}}^{2\left(\frac{M-3}{2}\right)}}\right]$$.

A well-known asymptotic expansion for the ratio of Gamma functions, for large of the argument $\:x$, is

18 $$\:\frac{{\Gamma\:}\left(x+a\right)}{{\Gamma\:}\left(x\right)}={x}^{a}\cdot\:\frac{1+a\left(a-1\right)}{2x}+\frac{a\left(a-1\right)\left(a-2\right)\left(3a-1\right)}{24{x}^{2}}+\dots\:$$.

In our case $\:x=\frac{M-3}{2}$ and $\:a=1/2$. Using the first two terms of this expansion we have

19 $$\:\frac{{\Gamma\:}\left(\frac{M-3}{2}+\frac{1}{2}\right)}{{\Gamma\:}\left(\frac{M-3}{2}\right)}\approx\:\sqrt{\frac{M-3}{2}}\cdot\:\frac{1+\frac{1}{2}\left(\frac{1}{2}-1\right)}{2\frac{M-3}{2}}+\frac{\frac{1}{2}\left(\frac{1}{2}-1\right)\left(\frac{1}{2}-2\right)\left(3\frac{1}{2}-1\right)}{24{\left(\frac{M-3}{2}\right)}^{2}}$$.

Simplifying leads to

20 $$\:\frac{{\Gamma\:}\left(\frac{M-2}{2}\right)}{{\Gamma\:}\left(\frac{M-3}{2}\right)}\approx\:\sqrt{\frac{M-3}{2}}-\frac{1}{4\sqrt{2\left(M-3\right)}}+\frac{1}{32\sqrt{{2\left(M-3\right)}^{3}}}$$.

Inserting this into (17) leads to

21 $$\:\mathrm{v}\mathrm{a}\mathrm{r}\left\{\widehat{RMS}\right\}\approx\:{\sigma\:}_{n}^{2}\cdot\:\left[\frac{M-3}{M}-\frac{2}{M}\cdot\:{\left(\sqrt{\frac{M-3}{2}}-\frac{1}{4\sqrt{2\left(M-3\right)}}+\frac{1}{32\sqrt{{2\left(M-3\right)}^{3}}}\right)}^{2}\right]$$.

This can be rewritten as

22 $$\:\mathrm{v}\mathrm{a}\mathrm{r}\left\{\widehat{RMS}\right\}\approx\:{\sigma\:}_{n}^{2}\cdot\:\left(\frac{M-3}{M}\right)\cdot\:\left[1-{\left(1-\frac{1}{4\cdot\:\left(M-3\right)}+\frac{1}{32\cdot\:{\left(M-3\right)}^{2}}\right)}^{2}\right]$$.

A good approximation that can be used for large number of samples is

23 $$\:\mathrm{v}\mathrm{a}\mathrm{r}\left\{\widehat{RMS}\right\}\approx\:\frac{{\sigma\:}_{n}^{2}}{2M}$$.

In the next section we will use numerical validation to verify the correctness of these expressions and to access how good are the approximations made in the more cumbersome expression given in Eq. (22) and in the simpler expression given in Eq. (23).

## Numerical validation

In order to check the correctness of the derivations presented in the previous section we will numerically simulate a set of data points sampled from a sinewave which is corrupted by additive noise. From these points we fit the best sinusoidal model that minimizes the square difference between the model and the data points, then compute the residuals, that is, the difference between the points and the model. From them we determine an estimate of the RMS value. By repeating this procedure a large number of times (*R*), we obtain a set of estimates that differ from one another due to the presence of additive noise. If there was no noise present, all estimated would have the same value. We then compute the variance of those estimates and a confidence interval for them.

The variance of the estimates has a chi-squared distribution with $\:n-1$ degrees of freedom where $\:n$ is the number of RMS estimates made. Note that this is not the number of sinusoidal data points used, $\:M$. The confidence intervals are given by

24 $$\:\left[(n-1)\frac{\mathrm{v}\mathrm{a}\mathrm{r}\left\{\widehat{RMS}\right\}}{{\chi\:}^{2}\left(1-\frac{\alpha\:}{2},n-1\right)};(n-1)\frac{\mathrm{v}\mathrm{a}\mathrm{r}\left\{\widehat{RMS}\right\}}{{\chi\:}^{2}\left(\frac{\alpha\:}{2},n-1\right)}\right]$$.

In this expression, *α* represents the significance level associated with the confidence interval. The corresponding confidence level is given by $\:1-\alpha\:$. For example, choosing α = 0.05 corresponds to a 95% confidence interval, and α = 0.01 corresponds to a 99% confidence interval.

**Fig. 1:**
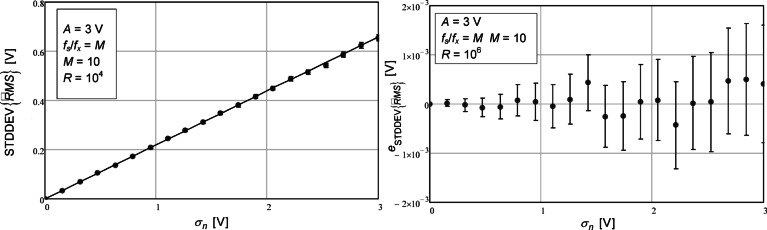
Standard deviation of the estimated RMS value as a function of the standard deviation of the additive noise (left). The circles represent the values obtained with the numerical simulations. The confidence intervals for a confidence level of 99.9% are represented by the vertical bars. The number of repetitions (*R*) was 10^4^. The solid line represents the value given by the theoretical expression (17). On the right, the difference between the values from the numerical simulation and theoretical expression are represented. The number of repetitions (*R*) in this case was 10^6^.

In the first simulation, we computed the standard deviation of the RMS estimated value for different amounts of additive noise standard deviation. The values obtained are represented in Fig. 1 (left) using circles and error bars. In the same figure we plotted, using a solid line, the analytical value given by the expression presented in Eq. (17). We chose to plot the standard deviation and not the variance because it depends linearly on the additive noise standard deviation and it is thus easier to verify the agreement. In all simulations carried out the initial phase was chosen randomly, uniformly distributed in the interval from 0 to 2p, in each repetition.

As seen in the left plot, there is excellent agreement between the values obtained from the numerical simulation and the ones given by the analytical expression. A sinusoidal signal with 3 V of amplitude and random initial phase was used from which a set of 10 data points (*M*) was sampled. This low value for the number of samples was chosen because the approximation made in the theoretical expression’s derivation is usually worst as the number of data points decreases; we therefore chose to show the results in an unfavorable condition and, at the same time, use the same number of data points in all simulation results presented here to facilitate comparison. In the case of the results presented on the left part of Fig. 1 the number of repetitions (*R*) used was 10^4^. Later on, we will present a plot where the number of samples used is varied.

The values depicted in Fig. 1 are shown in a tabular form in Table 1. The corresponding SNR values were computed as $\:10\cdot\:{\mathrm{l}\mathrm{o}\mathrm{g}}_{10}({A}^{2}/(2{\sigma\:}_{n}^{2}\left)\right)$.

**Table 1:** Standard deviation of the estimated RMS value as a function of the standard deviation of the additive noise. The second column has analytical values, and the third column has values obtained from the numerical simulation. The last column shows the relative error between both. The parameters are the same as in Fig. 1.

σₙ (V)	SNR (dB)	Simulated (V)	Analytical (V)	Relative Error (%)
0	∞	0	0	
0.158	22.565	0.035	0.035	−0.307
0.316	16.544	0.069	0.069	−0.057
0.474	13.022	0.105	0.104	−0.926
0.632	10.524	0.137	0.139	0.814
0.789	8.585	0.173	0.173	−0.019
0.947	7.002	0.208	0.208	−0.178
1.105	5.663	0.245	0.242	−1.243
1.263	4.503	0.279	0.277	−0.763
1.421	3.48	0.312	0.312	−0.052
1.579	2.565	0.349	0.346	−0.751
1.737	1.737	0.381	0.381	0.102
1.895	0.981	0.416	0.416	−0.123
2.053	0.286	0.449	0.45	0.198
2.211	−0.358	0.487	0.485	−0.432
2.368	−0.957	0.515	0.519	0.825
2.526	−1.518	0.543	0.554	2.11
2.684	−2.044	0.585	0.589	0.553
2.842	−2.541	0.624	0.623	−0.183
3	−3.01	0.654	0.658	0.656

In the right side of Fig. 1 one can see a plot with a difference between the numerically simulated values and the ones given by the analytical expression (17),

25 $$\:{e}_{STDDEV\left\{\widehat{RMS}\right\}}=\mathrm{S}\mathrm{T}\mathrm{D}\mathrm{D}\mathrm{E}\mathrm{V}\left\{\widehat{RMS}\right\}-\mathrm{S}\mathrm{T}\mathrm{D}\mathrm{D}\mathrm{E}\mathrm{V}{\left\{\widehat{RMS}\right\}}_{TEO}$$.

Note that all the error bars are around 0. Furthermore, the number of repetitions used in this simulation (*R*) was 10^6^. The sinusoidal parameters here and throughout this work were the same. The sinusoidal frequency ($\:{f}_{x}$) and the sampling frequency ($\:{f}_{s}$) were chosen so that the data points covered a single period of the sinusoidal signal,

26 $$\:{f}_{s}={f}_{x}\cdot\:M$$.

**Fig. 2:**
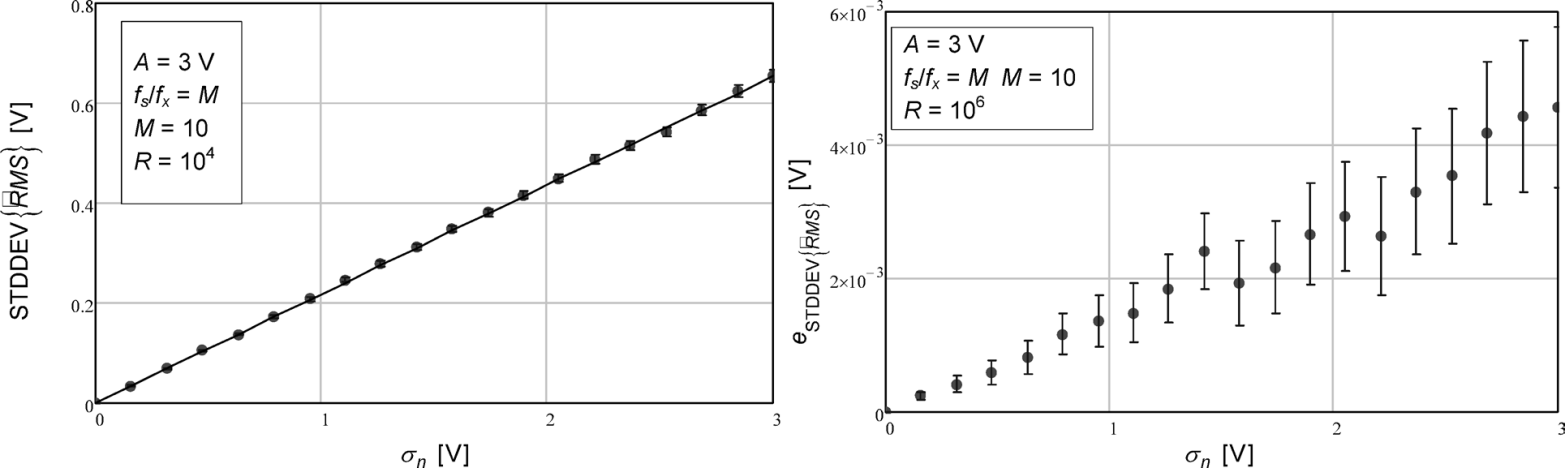
Standard deviation of the estimated RMS value as a function of the standard deviation of the additive noise (left). The circles represent the values obtained with the numerical simulations. The confidence intervals for a confidence level of 99% are represented by the vertical bars. The number of repetitions (*R*) was 10^4^. The solid line represents the value given by the theoretical expression (22). On the right, the difference between the values from the numerical simulation and theoretical expression are represented. The number of repetitions (*R*) in this case was 10^6^.

The next results are presented in Fig. 2 where the analytical expression used was the one given in (22). The same parameters were used in this simulation as the ones used in the simulation presented in Fig. 1. Note, in the left plot, the excellent agreement between error bars (numerical simulation results) and solid line (analytical results). In the right plot, where we have plotted the difference between the two, we observe also that the error bars are all around 0. Looking at the circles, however we can envision a small discrepancy since they are all below 0. This is to be expected since an approximation was made in (18) where the ratio of Gamma functions was approximated by the first two terms of an infinite series. This, however, has been shown here to be quite enough to achieve a very good approximate expression.

The analytical expression proposed in (22), although quite accurate, is a little cumbersome to use and would not be suitable if one wanted to determine the number of data points to use in order to satisfy an upper bound for the estimation uncertainty due to the non-linear relationship between RMS estimation variance and the number of samples. It would, however, be appropriate at the time of determining the confidence intervals for the estimated RMS value.

The values that are shown in Fig. 2 are shown in tabular form in Table 2.

**Table 2:** Standard deviation of the estimated RMS value as a function of the standard deviation of the additive noise. The second column has analytical values, and the third column has values obtained from the numerical simulation. The last column shows the relative error between both. The parameters are the same as in Fig. 2.

σₙ (V)	SNR (dB)	Simulated (V)	Analytical (V)	Relative Error (%)
0	∞	0	0	
0.158	22.565	0.035	0.035	1.641
0.316	16.544	0.069	0.071	1.896
0.474	13.022	0.105	0.106	1.01
0.632	10.524	0.137	0.141	2.784
0.789	8.585	0.173	0.177	1.935
0.947	7.002	0.208	0.212	1.773
1.105	5.663	0.245	0.247	0.687
1.263	4.503	0.279	0.282	1.176
1.421	3.48	0.312	0.318	1.901
1.579	2.565	0.349	0.353	1.188
1.737	1.737	0.381	0.388	2.058
1.895	0.981	0.416	0.424	1.829
2.053	0.286	0.449	0.459	2.157
2.211	−0.358	0.487	0.494	1.514
2.368	−0.957	0.515	0.53	2.795
2.526	−1.518	0.543	0.565	4.105
2.684	−2.044	0.585	0.6	2.518
2.842	−2.541	0.624	0.636	1.768
3	−3.01	0.654	0.671	2.623

**Fig. 3:**
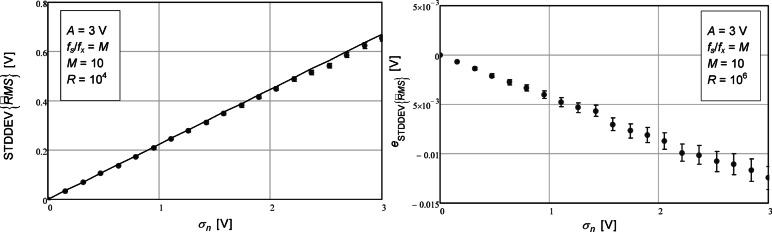
Standard deviation of the estimated RMS value as a function of the standard deviation of the additive noise (left). The circles represent the values obtained with the numerical simulations. The confidence intervals for a confidence level of 99% are represented by the vertical bars. The number of repetitions (*R*) was 10^4^. The solid line represents the value given by the theoretical expression (23). On the right, the difference between the values from the numerical simulation and theoretical expression are represented. The number of repetitions (*R*) in this case was 10^6^.

To make it easier to compute the number of data points to use, a third analytical expression was presented in Eq. (23). In Fig. 3 we observe that the agreement of the analytical expression with the numerically computed values is not as good as the two prevision expressions. In particular, the error, given by (25) is not null.

The values that are shown in Fig. 3 are shown in tabular form in Table 3.

**Table 3:** Standard deviation of the estimated RMS value as a function of the standard deviation of the additive noise. The second column has analytical values, and the third column has values obtained from the numerical simulation. The last column shows the relative error between both. The parameters are the same as in Fig. 3.

σₙ (V)	SNR (dB)	Simulated (V)	Analytical (V)	Relative Error (%)
0	∞	0.0000	0.0000	0.0
0.158	22.565	0.0506	0.0514	1.5
0.316	16.544	0.1012	0.1027	1.5
0.474	13.022	0.1486	0.1509	1.5
0.632	10.524	0.1992	0.2022	1.5
0.789	8.585	0.2498	0.2536	1.5
0.947	7.002	0.3004	0.3049	1.5
1.105	5.663	0.3510	0.3563	1.5
1.263	4.503	0.3984	0.4044	1.5
1.421	3.48	0.4490	0.4558	1.5
1.579	2.565	0.4996	0.5071	1.5
1.737	1.737	0.5502	0.5585	1.5
1.895	0.981	0.5977	0.6066	1.5
2.053	0.286	0.6483	0.6580	1.5
2.211	−0.358	0.6989	0.7093	1.5
2.368	−0.957	0.7495	0.7607	1.5
2.526	−1.518	0.8001	0.8121	1.5
2.684	−2.044	0.8475	0.8602	1.5
2.842	−2.541	0.8981	0.9116	1.5
3	−3.01	0.9487	0.9629	1.5

Using analytical expression (23) to determine the number of samples to use is quite simple:

27 $$\:M\ge\:\frac{{\sigma\:}_{n}^{2}}{2\cdot\:{B}_{RMS}^{2}}$$,

where $\:{B}_{RMS}$ is the upper bound set on the RMS estimation standard deviation.

**Fig. 4:**
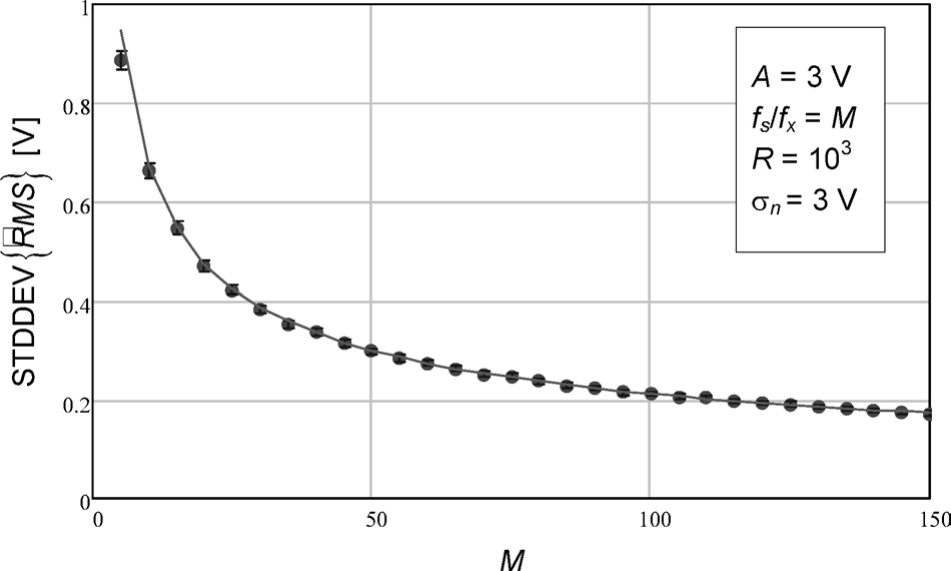
Standard deviation of the estimated RMS value as a function of the number of data points. The circles represent the values obtained with the numerical simulations. The confidence intervals for a confidence level of 99% are represented by the vertical bars. The number of repetitions (*R*) was 10^3^. The solid line represents the value given by the theoretical expression (23).

A final numerical simulation was carried out where the number of data points was varied. The results are shown in Fig. 4. Expression (23) was used for the analytical expression represented using the solid line. A very high value of additive noise standard deviation was used – it was as large as the sinusoidal amplitude. A good agreement is, however, still observed, especially for larger number of data points.

The results obtained using numerical simulations, and presented in this section, validate the correctness of the derivations made. More simulations were carried out under different conditions, and the agreement obtained was equally good. We chose to present here just the more relevant ones. All, however, validate the conclusions reached.

The simulations presented in this work were performed under the coherent sampling condition $\:{f}_{s}={f}_{x}\cdot\:M$, which ensures that the acquired samples correspond to an integer number of signal periods. This assumption is widely adopted because it simplifies the analysis and the least-squares estimation process. However, in practical measurement scenarios this condition is rarely satisfied, and the normalized angular frequency $\:{{\Omega\:}}_{0}=2\pi\:{f}_{x}/{f}_{s}$can take any value in the range $\:0<{{\Omega\:}}_{0}<\pi\:$. When sampling is non-coherent, slight spectral leakage and correlated residuals may appear, which can marginally modify the variance of the RMS estimator. Nevertheless, for moderate deviations from coherence, these effects are typically small and do not significantly affect the analytical expressions derived here.

In practice, when the stimulus frequency $\:{f}_{x}$is known, a three-parameter sine-fitting procedure is used to estimate amplitude, phase, and offset. When the frequency is unknown, a four-parameter sine-fitting method is typically employed. This approach iteratively refines the frequency estimate, and the final step—used to compute the residuals—is performed using the last and best value of the estimated frequency. Although this iterative procedure introduces a small additional dependency on $\:{{\Omega\:}}_{0}$, it primarily affects convergence speed rather than the underlying variance relationship derived in this paper. A detailed study of the influence of $\:{{\Omega\:}}_{0}$and frequency-estimation uncertainty on the residual statistics will be considered in future work.

## Conclusions

In this work, an analytical derivation of the variance of sinefitting residual RMS estimation was made. This estimation is carried out in the context of fitting a sinusoidal model to a set of data points. We considered that the data points are affected by additive noise, which, in real world conditions, is necessarily the case. The goal was to obtain a theoretical expression that relates the variance of the estimative with the standard deviation of the additive noise affecting the data points.

The principal novelty of this study is the derivation of an exact analytical model for the variance of the RMS residuals under additive noise—an aspect not previously covered by existing analytical or uncertainty models. This result extends earlier work on phase and timing noise, providing a more comprehensive foundation for uncertainty evaluation in sine-fitting–based measurement methods.

Three analytical expressions were presented, namely, Eq. (17), Eq. (22) and Eq. (23), each one with different degrees of accuracy and ease of use. The first one, Eq. (17), involved the use of the Gamma function. In this case no approximation was made but the fact that it uses the Gamma function which does not have a general closed-form expression in terms of elementary functions like polynomials, exponentials, logarithms, trigonometric functions, etc., makes it a little harder to use.

The second expression derived, Eq. (22), used the first two terms of a series approximation to the Gamma function. In this case it was indeed possible to derive a closed form expression for the variance of the residuals of sinefitting. This expression can be used to compute the confidence intervals for the estimates made, if the amount of additive noise present is known. It is, however, not feasible to use it to determine the number of data points that should be used in order to attain a prespecified bound on the estimation uncertainty, because of its nonlinear dependence on the number of data points.

A third expression was thus proposed which is very simple, namely, Eq. (23), but whose agreement with the values obtained through numerical simulations is not perfect. It is, however, quite good and can be used in practice in most cases.

Future work might address the variance of this same estimator under different non-ideal conditions like the presence of phase noise, jitter or quantization error. One might also want to study if this estimator is biased in any conditions. Furthermore, a study of this estimator is going to be carried out in the specific condition where the stimulus frequency is not known and has to be estimated before using the three-parameter sinefitting to estimate the residuals.

## Data Availability

All data generated or analysed during this study are included in this published article.
